# Effects of Bu Shen Yi sui capsule on NogoA/NgR and its signaling pathways RhoA/ROCK in mice with experimental autoimmune encephalomyelitis

**DOI:** 10.1186/s12906-017-1847-4

**Published:** 2017-07-01

**Authors:** Ling Fang, Yongqiang Wang, Qi Zheng, Tao Yang, Peiyuan Zhao, Hui Zhao, Qiuxia Zhang, Yuanyuan Zhao, Fang Qi, Kangning Li, Zhenzhen Chen, Junling Li, Nan Zhang, Yongping Fan, Lei Wang

**Affiliations:** 1 School of Traditional Chinese Medicine, Beijing Key Lab of TCM Collateral Disease Theory Research, Capital Medical University, Beijing, 100069 People’s Republic of China; 2 Beijing Tian Tan Hospital, Capital Medical University, Beijing, 100050 People’s Republic of China; 3 Daxing Hospital Affiliated to Capital Medical University, Beijing, 102600 People’s Republic of China; 4 Guang An Men Hospital of China Academy of Chinese Medical Sciences, Beijing, 100053 People’s Republic of China; 5 Core Facility Center, Capital Medical University, Beijing, 100069 People’s Republic of China

**Keywords:** Experimental autoimmune encephalomyelitis, Multiple sclerosis, Bu Shen Yi sui capsule, PGP9.5, P-tau, Gap-43, KI67, Nestin, NogoA/NgR, RhoA/ROCK

## Abstract

**Background:**

Axon growth inhibitory factors NogoA/Nogo receptor (NgR) and its signaling pathways RhoA/Rho kinase (ROCK) play a critical role in the repair of nerve damage in multiple sclerosis (MS). Bu Shen Yi Sui Capsule (BSYSC) is an effective Chinese formula utilized to treat MS in clinical setting and noted for its potent neuroprotective effects. In this study, we focus on the effects of BSYSC on promoting nerve repair and the underlying mechanisms in mice with experimental autoimmune encephalomyelitis (EAE), an animal model of MS.

**Methods:**

The EAE mouse model was induced by injecting subcutaneously with myelin oligodendrocyte glycoprotein (MOG) _35–55_ supplemented with pertussis toxin. BSYSC was orally administrated at dose of 3.0 g/kg once a day for 40 days. The levels of protein gene product (PGP) 9.5, p-Tau, growth associated protein (GAP) -43, KI67 and Nestin in the brain or spinal cord on 20 and 40 day post-induction (dpi) were detected via immunofluorescence and Western blot analysis. Furthermore, NogoA/NgR and RhoA/ROCK signaling molecules were studied by qRT-PCR and Western blot analysis.

**Results:**

Twenty or 40 days of treatment with BSYSC increased markedly PGP9.5 and GAP-43 levels, reduced p-Tau in the brain or spinal cord of mice with EAE. In addition, BSYSC elevated significantly the expression of KI67 and Nestin in the spinal cord 40 dpi. Further study showed that the activation of NogoA/NgR and RhoA/ROCK were suppressed by the presence of BSYSC.

**Conclusions:**

BSYSC could attenuate axonal injury and promote repair of axonal damage in EAE mice in part through the down-regulation of NogoA/NgR and RhoA/ROCK signaling pathways.

## Background

Multiple sclerosis (MS) is a central nervous system (CNS) autoimmune mediated inflammatory neurodegenerative disease [1], which is characterized by pathologic inflammation, demyelination, axonal degeneration and neuronal injury [2–4]. Recently, a large number of epidemiological studies confirm that annual global incidence and prevalence rate of MS increase progressively over the past years. MS predominantly occurs in young women, manifested with damages in the brain, spinal cord and the optic nerve, which eventually leads to sensory dysfunction, motor function disability and blindness in patients, even at early disease stages [5]. Experimental autoimmune encephalomyelitis (EAE) is very similar with human MS in pathological changes which is widely recognized as an animal model in MS research [6].

Growing evidence showed that demyelination and axonal damage is the major cause of neurological disability in MS patients, especially early occur axonal damage in the disease [7, 8]. Therefore, the treatment of axonal repair is of great clinical significance in MS. With severe axonal injury in brain and spinal cord occurs in MS/EAE, the self repair function is activated to partially restore neurological function through a certain way in the early stage of axonal injury [9]. However, the natural recovery is far from enough to compensate for damaged axonal function. The research found that axon growth inhibitory factors play an important role in the failure of axonal regeneration. The inhibitors such as NogoA, myelin-associated glycoprotein (MAG) and oligodendrocyte myelin glycoprotein (OMgp) bind to a common Nogo-66 receptor (NgR) [10]. NgR signaling requires a p75 (NTR) or TROY in combination with an adaptor LINGO-1. Thus, each of the inhibitors interacts in NgR and its co-receptors to transduce the inhibitory signal into neurons [11], activating guanosine triphosphatase (GTPase), increasing the activity of the neuronal cytoskeleton regulatory factor RhoA, resulting in neuronal growth cone collapse and inhibiting the extension and regeneration of axons [12–15].

Since the precise pathogenesis of MS is unclear, there is no safe and effective therapeutics yet [16]. Traditional Chinese medicine (TCM) in treatment for MS gives promising results [17, 18]. In addition to the previous studies, we found that BSYSC had a good effect in the treatment of MS [19, 20]. The neuroprotective effect of BSYSC was found in EAE animal model [21–23]. However, the mechanism was not understood. In this study, the effects of BSYSC on axonal repair and regulation of axon growth inhibitory factors NogoA/NgR and its signaling pathways RhoA/ROCK in EAE mice were explored. The results revealed a molecular mechanism, at least in partial, of BSYSC on axonal regeneration, and provided scientific evidence supporting its clinical application in treatment of MS.

## Methods

### Animals

The experimental protocol was approved by the Ethics Committee of Capital Medical University (No. 2011-X-001). Specific pathogen-free (SPF) grade female C57BL/6 mice weighting 16–18 g, 6-to-8-week-old, were purchased from Beijing Vital River Laboratories, China [certification NO. SCXK (JING) 2006–0009]. The mice were kept in the Center of Laboratory Animals at Capital Medical University [certification NO. SYXK (JING) 2010–0020]. The mice were housed under a 12-h light/dark cycle in individual ventilated cages and maintained in a SPF grade environment.

### BSYSC preparation

BSYSC consisted of ten crude herbs including *Rehmanniae Radix*, *Rehmanniae radix Praeparata*, *Polygoni Multiflori Radix,Rhei Radix et Rhizoma*, *Leonuri Herba, Fritillariae Thunbergii Bulbus*, *Hirudo*, *Scorpio*, *Gastrodiae Rhizoma* and *Forsythiae Fructus* at a ratio of 10:10:10:2:10:6:3:2:3:6. All of the herbal medicines in BSYSC were originally obtained from Beijing Ya Dong Biological Pharmacy Co., Ltd. (Beijing, China). Validation specimens were deposited at the Brain Disease Laboratory of the School of Traditional Chinese Medicine, Capital Medical University. The crude herbs were identified by associate Prof. Rong Luo in accordance with the Pharmacopoeia of People’s Republic of China 2015 (1st set) [24]. The main components and chemical characteristic fingerprinting of BSYSC were identified by ultra-performance liquid chromatography-quadrupole time-of-flight mass spectrometry (UPLC-QTOF-MS). The contents of acteoside and forsythiaside were detected, which were not less than 0.7 mg/g and 0.6 mg/g, respectively, the same batch of BSYSC was used in the experiment. The detailed preparation procedure and quality control of BSYSC has been described in our recent report [23].

### Drugs and reagents

Prednisone acetate (PA) was purchased from Zhejiang Xianju Pharmaceutical Co., Ltd. (Zhejiang, China). Myelin oligodendrocyte glycoprotein (MOG) _35–55_ (MEVGWYRSPFSRVVHLYRNGK, purity was >95%) was synthesized by Beijing SciLight Biotechnology Co., Ltd. (Beijing, China). Mycobacterium tuberculosis H37Ra was purchased from Difco Co., Ltd. (Franklin Lakes, NJ, USA). Complete Freund’ adjuvant (CFA) and pertussis toxin (PTX) were purchased from Sigma-Aldrich (St. Louis, MO, USA). Protein gene product (PGP) 9.5, Tau (phospho S262) and growth associated protein (GAP) -43, KI67 rabbit anti-mouse antibodies were purchased from Abcam Co., Ltd. (Cambridge, UK), protein Nestin mouse anti-mouse antibodies were purchased from Abcam Co., Ltd. (Cambridge, UK). NogoA rabbit anti- mouse antibody was purchased from Abcam Co., Ltd. (Cambridge, UK). NgR was purchased from Millipore Co., Ltd. (Massachusetts, USA). RhoA was purchased from Cell Signaling Technology Co., Ltd. (Boston, USA). ROCKII was from Abcam Co., Ltd. (Cambridge, UK). Alexa Fluor 488-labeled goat anti-rabbit, goat anti-mouse and 594-labeled goat anti-rabbit IgG (H + L) antibody were purchased from Jackson Co., Ltd. (Jackson, USA), GAPDH goat anti-rabbit IgG (H + L) antibody was purchased from Abcam Co., Ltd. (Cambridge, UK). The detailed contents are shown in Table 1. Western blot (WB) kits were purchased from Applygen Technologies Inc. (Beijing, China). Real-time quantitative reverse transcription-polymerase chain reaction (qRT-PCR) kits and reverse transcription kits were purchased from Tiangen Biotech Co., Ltd. (Beijing, China). PCR primers were synthesized by TaKaRa Biotechnology Co. Ltd. (Dalian, China).

**Table 1:** The antibodies used in the experiment

Name	Host	Dilution	Company
PGP 9.5	Rabbit	IF: 1:300 WB: 1:80000 (brain); 1:50000 (spinal cord)	Abcam
p-Tau	Rabbit	IF: 1:50 WB: 1:20000 (brain); 1:10000 (spinal cord)	Abcam
GAP-43	Rabbit	WB: 1:4000	Abcam
KI67	Rabbit	IF: 1: 100	Abcam
Nestin	Mouse	IF: 1: 100	Abcam
NogoA	Rabbit	WB: 1:20000 (brain); 1:2500 (spinal cord)	Abcam
NgR	Rabbit	WB: 1:2000 (brain); 1:5000 (spinal cord)	Millipore
RhoA	Rabbit	WB: 1:20000 (brain); 1:5000 (spinal cord)	Cell Signaling
ROCK	Rabbit	WB: 1:5000	Abcam
GAPDH	Goat	WB: 1:2000	Abcam

### Induction and treatment of EAE

Mice were randomly divided into four groups: normal control (NC, *n* = 20), EAE model (MO, *n* = 20), EAE + PA-treated (PA, *n* = 20) and EAE + BSYSC-treated (BSYSC, *n* = 20). The mice with EAE were immunized subcutaneously with 50 μg of MOG_35–55_ emulsified in CFA containing 400 μg MTB. PTX (500 ng) was followed by intraperitoneal injection on 0 and 2 day post-induction (dpi) [25]. The same volume of PBS was injected into a parallel group of mice as NC. For treatment of mice with medicines, mice in BSYSC group were given orally 3.0 g/kg BSYSC once a day for 40 days. The dose of BSYSC was more effective against EAE in our previous study [22–24]. Mice in PA group were administered orally PA at a dose of 6 mg/kg after the EAE onset (the ninth day). Mice in NC and EAE groups were treated with equal amounts of distilled water.

### Sample collection

The mice were sacrificed ﻿20 dpi (acute stage, neurological function scores at a peak) and 40 dpi (remission stage, no further increase in the signs of EAE). The brain and spinal cord of 5 mice was stored in 4% paraformaldehyde to be used for immunofluorescence (IF) analysis, and the brain and spinal cord of another 5 mice were immediately frozen for qRT-PCR and Western blot analyses.

### IF analysis

The brain and spinal cord of mice were formalin-fixed, conventionally dehydrated, embedded in paraffin, and cut into serial sections. The sections were dewaxed, and boiled with citric acid at 95 °C for 20 min. Then the sections were cooled to 30 °C, and blocked with 10% goat serum at 37 °C for 60 min. Subsequently the sections were incubated with primary detection antibody [rabbit anti-mouse PGP9.5 (1:300 dilution), rabbit anti-mouse p-Tau (1:50 dilution), rabbit anti-mouse KI67 (1:100 dilution), mouse anti-mouse Nestin (1:100 dilution)] at 4 °C for 48 h. After 37 °C rewarming for 60 min, the sections were incubated with the biotin-labeled secondary antibody [Alexa Fluor 488-labeled goat anti-rabbit IgG (H + L), revealing PGP9.5 (1:1000 dilution), p-Tau (1:400 dilution), goat anti-mouse IgG (H + L), revealing Nestin(1:400 dilution) and Alexa Fluor 594-labeled goat anti-rabbit IgG (H + L), revealing KI67 (1:400 dilution)] at 37 °C for 60 min, then mounted with DAPI-Fluoromount-G, and stored at 4 °C. Finally, the sections were observed under a Leica DM4000B fluorescence microscope (Leica, Solms, Germany). The image analysis was performed using Leica Qwin analysis software (Leica). Quantitative analysis was carried out with a NIS-Elements BR 3.0 system. Five high-power fields (×400) were selected in each sample and positive results were expressed as integrated optical density (IOD) values.

### QRT-PCR analysis

NogoA, NgR, RhoA and ROCK mRNA expression in brain and spinal cord of mice were observed by qRT-PCR. In short, total RNA from approximately 30 mg tissues was extracted according to the manufacturer’s instructions. The cDNA was obtained from total RNA by the reverse transcription of 1 μg of total RNA by the reverse transcription kit. The PCRs were performed with the following reaction protocol: 95 °C for 15 min, followed by 40 cycles of denaturation at 95 °C for 30 s, annealing at 54 °C for 10 s and extension at 72 °C for 10 s (Applied Biosystems 7300, Foster City, CA, USA). The relative quantification (RQ) was analyzed using the 2^-ΔΔCt^ method. The sequences of primers were designed for RT-PCR using GenBank Accession sequences as follows: NogoA forward, 5′-CTTGGTCATGTGAACAGCACAATAA-3′, reverse, 5′-CATTGAACAAGGCACCAACGTAA-3′; NgR forward, 5′-TCCAGTCATGCCGAAATCTCAC-3′, reverse, 5′-TGGTAGGGTCCACGACATGAAG-3′; RhoA forward, 5′-GGAGTGTTCAGCAAAGACCAAAG-3′, reverse, 5′-CACAAGATGAGGCACCCAGA-3′; ROCK forward, 5′-GGTATCGTCACAAGTAGCAGCATCA-3′, reverse, 5′-TAAACCAGGGCATCCAATCCA-3′;β-actin forward, 5′-CATCCGTAAAGACCTCTATGCCAAC-3′, reverse, 5′-ATGGAGCCACCGATCCACA-3′. β-actin mRNA served as an internal control. The amplified fragments were 124, 144, 111, 140 and 171 base pairs (bp), respectively.

### Western blotting analysis

Protein expressions of PGP9.5, p-Tau, GAP-43, NogoA, NgR, RhoA and ROCK in brain and spinal cord of mice were detected by Western blot according to standard protocols. Briefly, 20 μg proteins in each sample were processed by 5% and 10% SDS-PAGE and transferred onto a 0.45 μm polyvinylidene fluoride membrane (Millipore, USA). The membranes were incubated with primarily anti-PGP9.5 antibody (brain 1:80,000, spinal cord 1:50,000 dilution), anti-p-Tau antibody (brain 1:20,000, spinal cord 1:10,000 dilution), anti-GAP-43 antibody (1:4000 dilution), anti-NogoA antibody (brain 1:20,000, spinal cord 1:2500 dilution), anti-NgR antibody (brain 1:2000, spinal cord 1:5000 dilution), anti-RhoA antibody (brain 1:20,000, spinal cord 1:5000 dilution), anti-ROCK antibody (1:5000 dilution), or anti-GAPDH antibody (1:2000 dilution) as controls, followed by incubation with secondary goat anti-rabbit IgG (1:20,000) and electrochemiluminescence (ECL) reagent for 1 min. The membrane was exposed to Kodak film (Japan). Data were expressed by the integrated optical density (IOD) ratio using Image J (National Institutes of Health, USA).

### Statistical analysis

The data were represented as means ± standard error (SE). The data with normally distributed and equal variances were examined using one-way ANOVA with a post-hoc LSD test. Otherwise, the data were performed thyc=10?>rank-sum test. The family-wise error rate was controlled by the statistical method of Bonferroni. The differences were considered significant at a value of *p* < 0.05.

## Results

### Effects of BSYSC on the protein expression of PGP9.5 in EAE mice

**Fig. 1:**
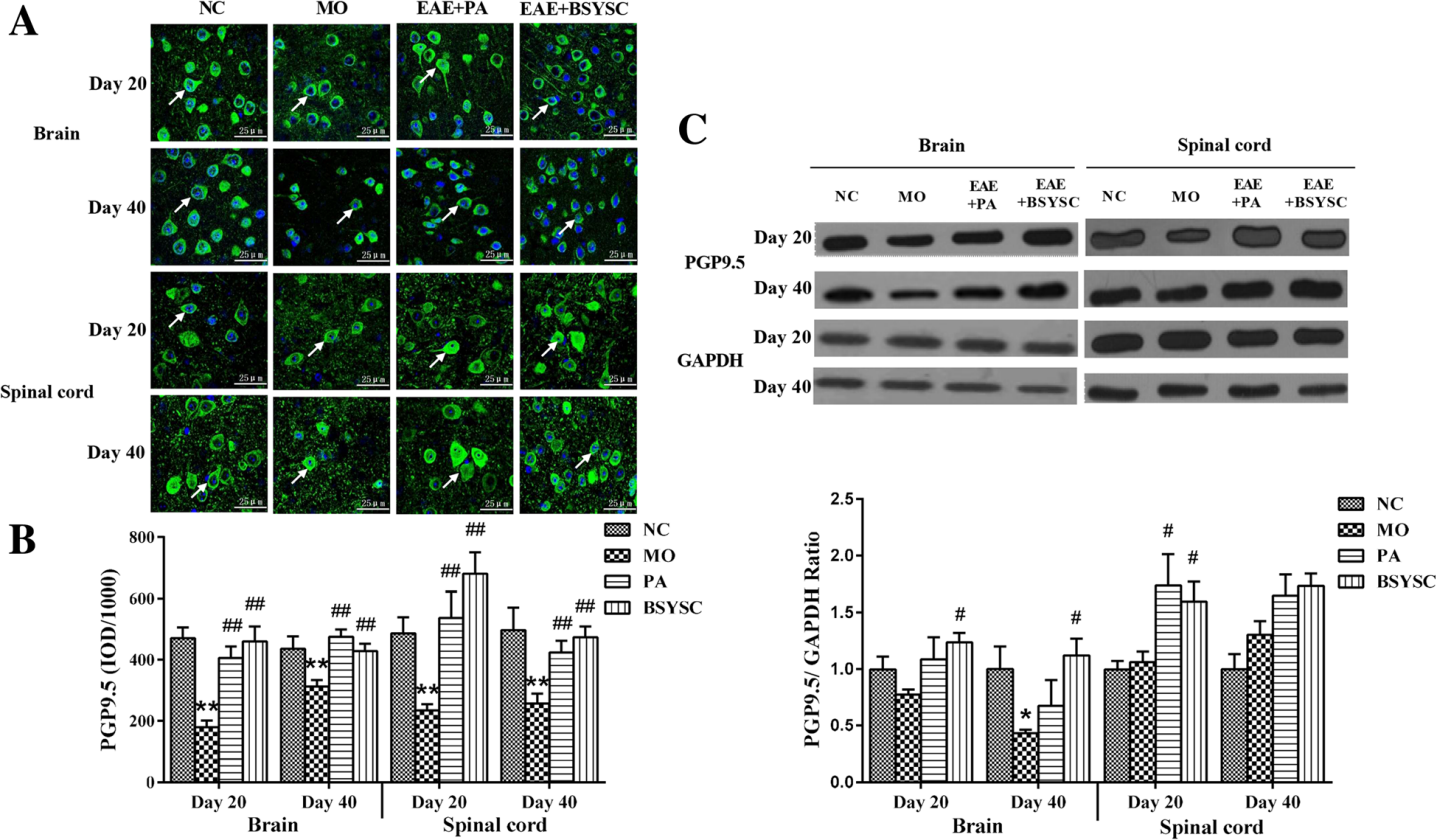
PGP9.5 protein expression in the brain and spinal cord of mice treated with or without BSYSC 20 and 40 dpi. An increase with different levels in PGP9.5 protein was found by treatment of BSYSC. **a** The representative immunofluorescent staining of PGP9.5 was shown in each group, the white arrows indicate the positive regions, magnification (×40). **b** Semi-quantification of integrated optical density (IOD) on PGP9.5 expression was exhibited. **c** The protein level of PGP9.5 was measured by Western blot and quantified using GAPDH as the loading controls. Data are expressed as means ± SE, *n* = 5 for each group. * *p* < 0.05, ** *p* < 0.01vs. NC; # *p* < 0.05, ## *p* < 0.01 vs. MO

IF analysis showed that PGP9.5 protein expression in the brain and spinal cord 20 and 40 dpi was reduced significantly in MO mice as compared with the NC mice (*p* < 0.01). The treatment with PA and BSYSC increased markedly PGP9.5 levels in the densitometry quantification as compared with the MO mice (*p* < 0.01, Fig. 1a, b). Western blot analysis demonstrated that PGP9.5 protein in the brain 40 dpi was only reduced markedly in the MO mice as compared with the NC mice (*p* < 0.05). A significant increase in PGP9.5 protein of the brain 20 and 40 dpi was observed in BSYSC mice as compared with the MO mice (*P <* 0.05). The increased PGP9.5 in the spinal cord 20 dpi was also shown in both PA and BSYSC mice as compared with the MO mice (*p* < 0.05, Fig. 1c).

### Effects of BSYSC on the protein expression of p-Tau in EAE mice

**Fig. 2:**
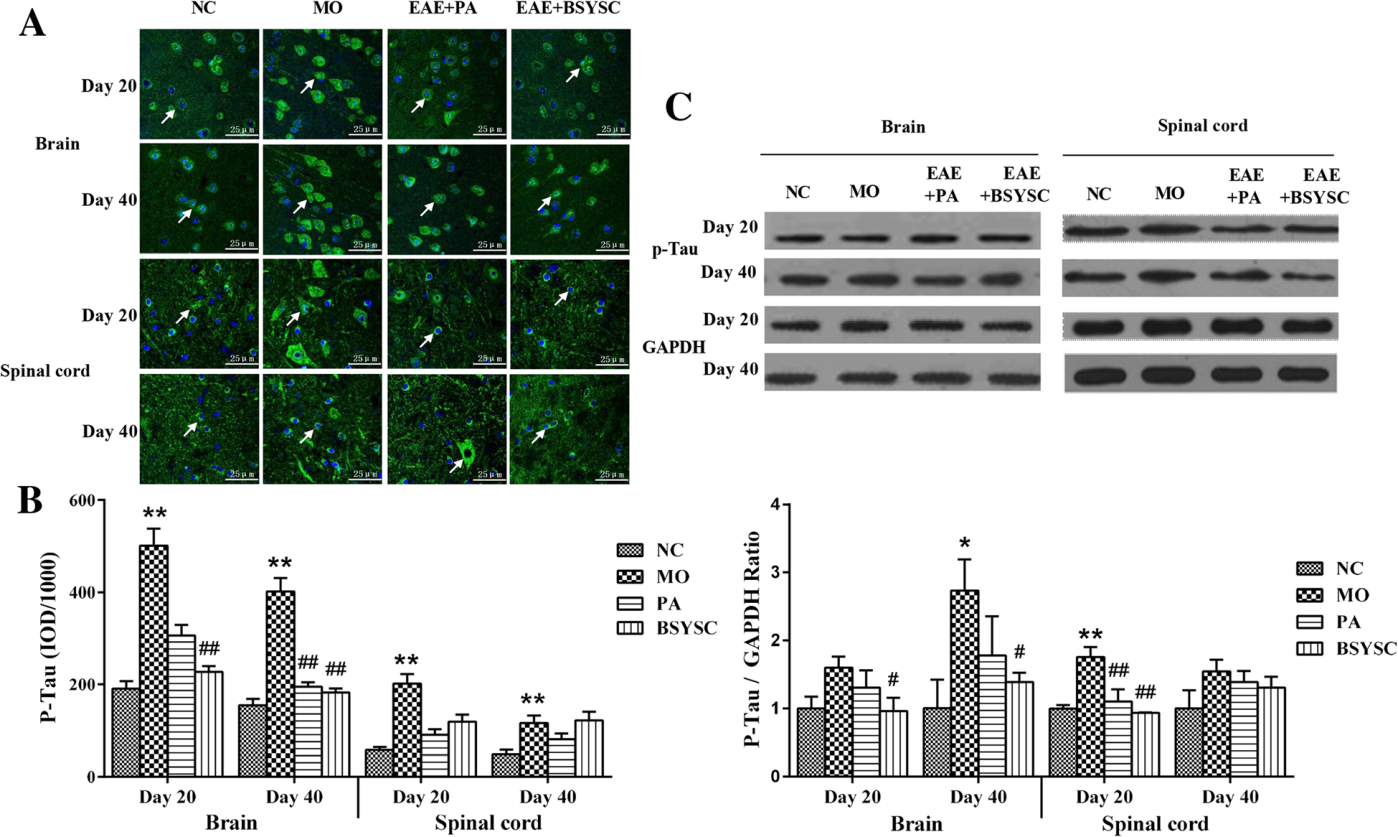
P-Tau protein expression in the brain and spinal cord of mice treated with or without BSYSC 20 and 40 dpi. The protein levels of p-Tau were reduced to different degrees in BSYSC group. **a** The representative immunofluorescent staining of p-Tau was shown in each group, the white arrows indicate the positive regions, magnification (×40). **b** Semi-quantification of integrated optical density (IOD) on p-Tau expression was exhibited. **c** The protein level of P-Tau was measured by Western blot and quantified using GAPDH as the loading controls. Data are expressed as means ± SE, *n* = 5 for each group. * *p* < 0.05, ** *p* < 0.01 vs. NC; # *p* < 0.05, ## *p* < 0.01 vs. MO

In the IF analysis, a significant increase on p-Tau protein expression in the brain and spinal cord 20 and 40 dpi in MO mice as compared with NC mice (*p* < 0.01). However, p-Tau protein in the brain 20 dpi was reduced significantly in BSYSC mice as compared with the MO mice (*p* < 0.01), and it also obviously reduced in the brain 40 dpi in both PA and BSYSC mice (*p* < 0.01). While no significant difference in p-Tau protein of the spinal cord 20 and 40 dpi in both PA and BSYSC mice (Fig. 2a, b). Western blot analyses showed that p-Tau at the protein levels appeared a significant increase in the brain 40 dpi and the spinal cord 20 dpi in MO mice as compared with NC mice (*p* < 0.05 or *p* < 0.01). Whereas a significant reduction in p-Tau protein of the brain 20 and 40 dpi was observed in BSYSC mice as compared with the MO mice (*p* < 0.05), and down-regulation of p-Tau protein of spinal cord 20 dpi was also shown in both PA and BSYSC mice (*p* < 0.01, Fig. 2c).

### Effects of BSYSC on the protein expression of GAP-43 in EAE mice

**Fig. 3:**
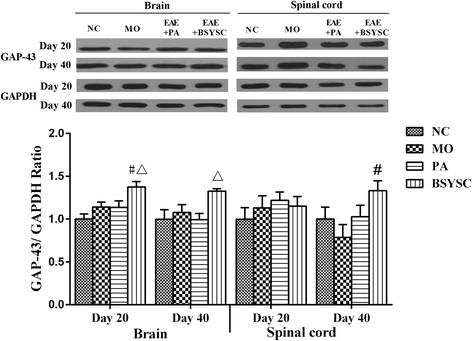
GAP-43 protein expression in the brain and spinal cord of mice treated with or without BSYSC 20 and 40 dpi. A significant increase in GAP-43 protein was found in the brain 20 dpi and in the spinal cord 40 dpi in BSYSC group. The protein level of GAP-43 was measured by Western blot and quantified using GAPDH as the loading controls. Data are expressed as means ± SE, *n* = 5 for each group. # *p* < 0.05 vs. MO; △ *p* < 0.05 vs. PA

No significant difference on GAP-43 protein expression was found between the NC and MO mice in western blot analysis. GAP-43 protein in the brain 20 dpi and the spinal cord 40 dpi was increased in BSYSC mice as compared with the MO mice (*P* < 0.05). The increase of GAP-43 protein of the brain 20 and 40 dpi in BSYSC mice appeared to be larger than that of PA mice (*P* < 0.05, Fig. 3).

### Effects of BSYSC on the protein expression of KI67 and Nestin in EAE mice

**Fig. 4:**
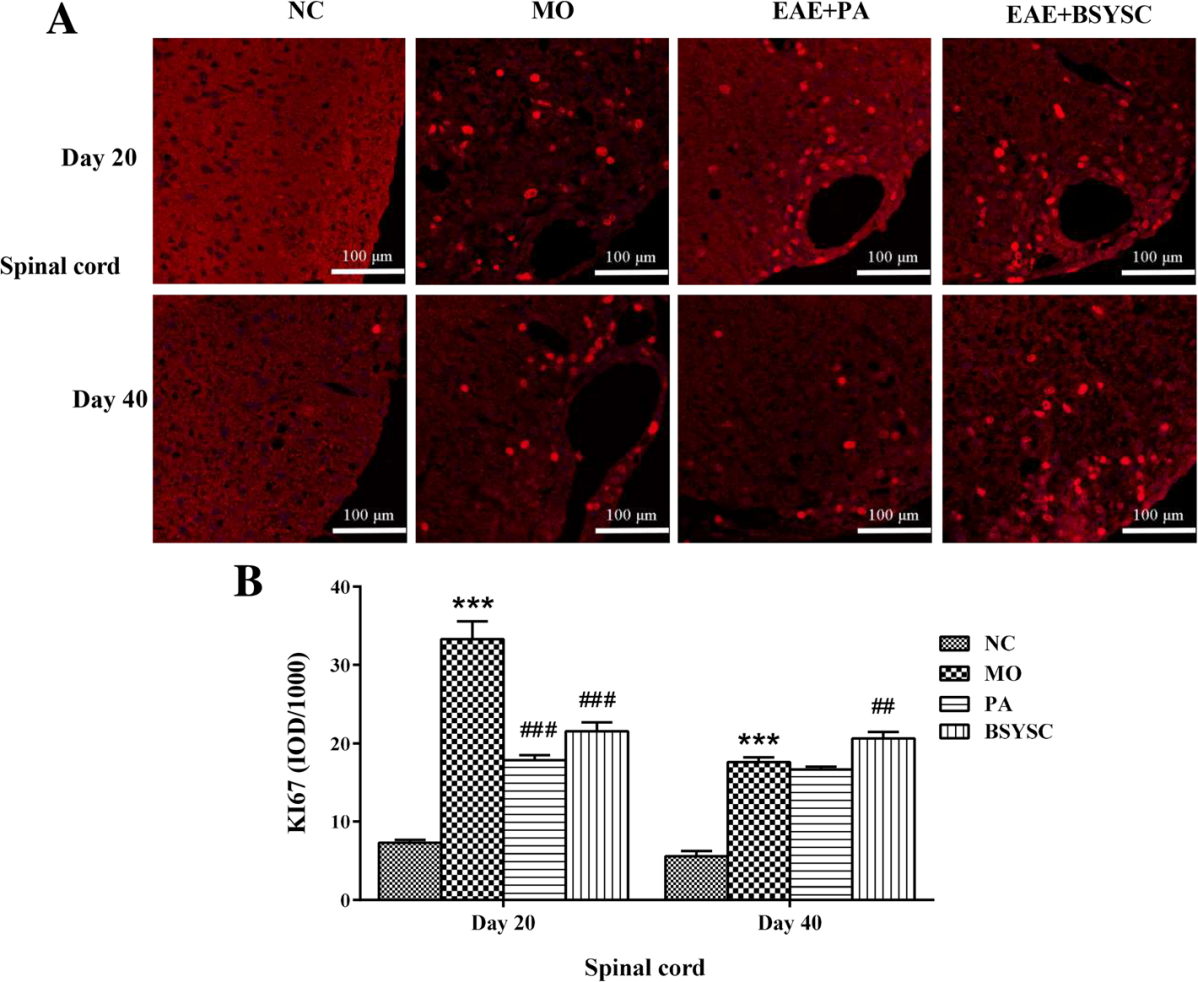
KI67 protein expression in the spinal cord of mice treated with or without BSYSC 20 and 40 dpi. BSYSC significantly decreased KI67 expression 20 dpi, but increased KI67 40 dpi. **a** The representative immunofluorescent staining of KI67 was shown in each group, magnification (×40). **b** Semi-quantification of integrated optical density (IOD) on KI67 expression was exhibited. Data are expressed as means ± SE, *n* = 5 for each group. *** *p* < 0.001 vs. NC; ## *p* < 0.01, ### *p* < 0.001 vs. MO

**Fig. 5:**
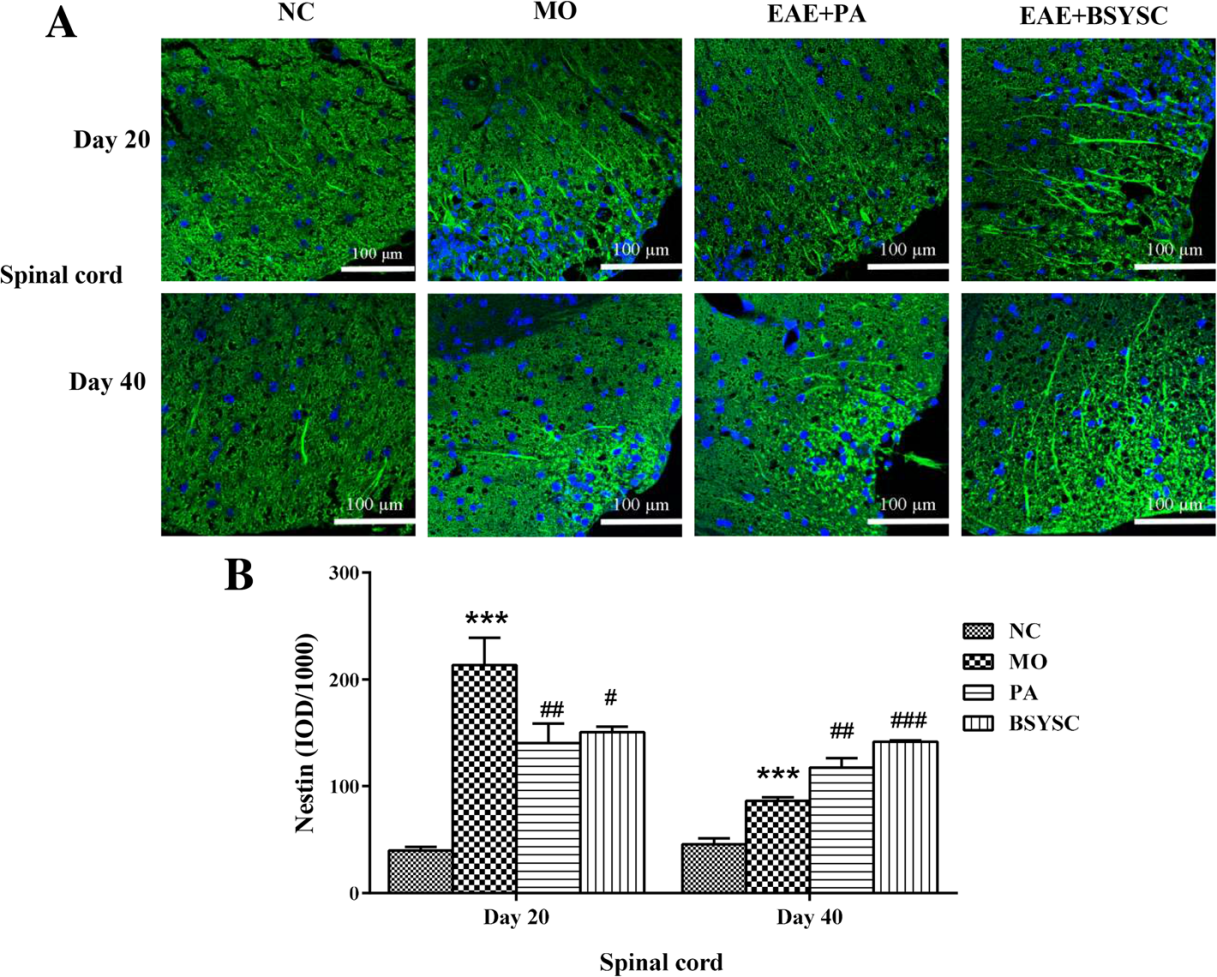
Nestin expression in the spinal cord of mice treated with or without BSYSC 20 and 40 dpi. A significant decreased Nestin expression was found in the spinal cord of 20 dpi in BSYSC mice, but increased Nestin was found 40 dpi. **a** The representative immunofluorescent staining of Nestin was shown in each group, magnification (×40). **b** Semi-quantification of integrated optical density (IOD) on Nestin expression was exhibited. Data are expressed as means ± SE, *n* = 5 for each group. *** *p* < 0.01 vs. NC; # *p* < 0.05, ## *p* < 0.01, ### *p* < 0.001 vs. MO

The fluorescent imaging analysis showed that KI67 and Nestin protein expression in the spinal cord 20 and 40 dpi in the densitometry quantification was increased significantly in MO mice as compared with the NC mice (*P* < 0.001). Compared with the MO mice, KI67 and Nestin levels 20 dpi was reduced markedly by treatment with PA and BSYSC (*P* < 0.001or *P* < 0.01 or *P* < 0.05), a significant increase in KI67 and Nestin 40 dpi was observed in BSYSC or PA mice (*P <* 0.01 or *P* < 0.001, Fig. 4a, b and Fig. 5a, b).

### Effects of BSYSC on the mRNA and protein expressions of NogoA/NgR in EAE mice

**Fig. 6:**
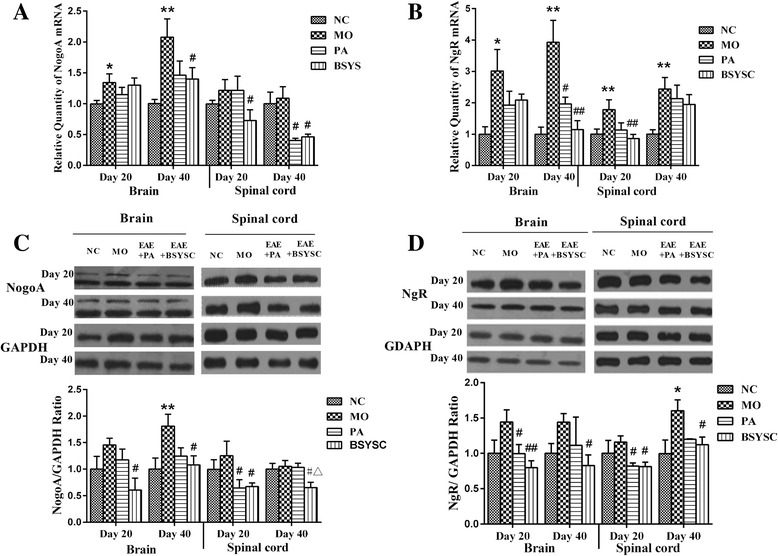
Expressions of mRNA and protein of NogoA and NgR in the brain and spinal cord of mice treated with or without BSYSC 20 and 40 dpi. Expression of NogoA and NgR mRNA and protein had suffered different degrees of decrease in BSYSC group. The mRNA levels of NogoA (**a**) and NgR (**b**) were detected by qRT-PCR analyses. The protein levels of NogoA (**c**) and NgR (**d**) were measured by Western blot and quantified using GAPDH as the loading controls. Data are expressed as means ± SE, *n* = 5 for each group. * *p* < 0.05, ** *p* < 0.01 vs. NC; # *p* < 0.05, 5, ## *p* < 0.01 vs. MO; △*p* < 0.05 vs. PA

The qRT-PCR results showed that NogoA mRNA in brain 20 and 40 dpi significantly increased in the MO mice as compared with the NC mice (*P <* 0.05 or *P <* 0.01), whereas the levels of NogoA mRNA were decreased significantly in the brain 40 dpi and the spinal cord 20 dpi in BSYSC mice as compared with the MO mice (*P <* 0.05), and it was also decreased in spinal cord 40 dpi in both PA and BSYSC mice (*P <* 0.05, Fig. 6a). Compared with the NC mice, NgR mRNA was significantly increased in brain and spinal cord 20 and 40 dpi in the MO mice as compared with the NC mice (*P <* 0.05 or *P <* 0.01). Decreased amounts of NgR mRNA were found in brain 40 dpi in both PA and BSYSC mice as compared with the MO mice(*P <* 0.05 or *P <* 0.01), and NgR mRNA it was also decreased in the spinal cord 20 dpi in BSYSC mice (*P <* 0.01, Fig. 6b).

Western blot analyses showed that NogoA protein was increased markedly, specifically in brain 40 dpi in the MO mice as compared with the NC mice (*P <* 0.01). Decreased NogoA levels were observed in brain and spinal cord 20 and 40 dpi in BSYSC mice (*P <* 0.05), and it also decreased in spinal cord 20 dpi in PA mice (*P <* 0.05). In addition, BSYSC treatment seemed to perform better than PA as in the result of spinal cord 40 dpi (*P <* 0.05, Fig. 6c). NgR was increased significantly at the protein level in spinal cord 40 dpi as compared with the NC mice (*P <* 0.05). A significant reduction of NgR protein in brain and spinal cord 20 dpi was observed in both PA and BSYSC mice as compared with the MO mice (*P <* 0.05 or *P <* 0.01), and NgR protein was also decreased in the brain and spinal cord 40 dpi in BSYSC mice (*P <* 0.05, Fig. 6d).

### Effects of BSYSC on mRNA and the protein expressions of RhoA and ROCK in EAE mice

**Fig. 7:**
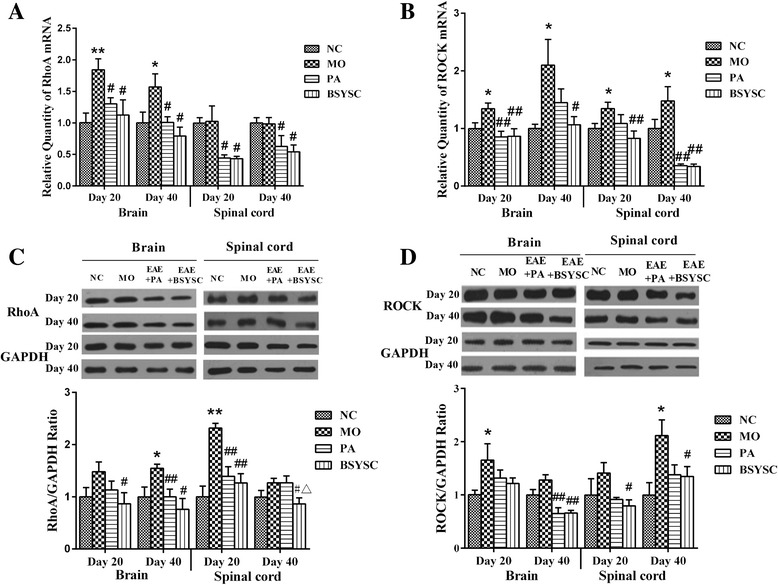
Expressions of mRNA and protein of RhoA and ROCK in the brain and spinal cord of mice treated with or without BSYSC 20 and 40 dpi. RhoA and ROCK mRNA and protein levels were decreased to some extent by treatment of BSYSC. The mRNA levels of RhoA (**a**) and ROCK (**b**) were detected by qRT-PCR analyses. The protein levels of RhoA (**c**) and ROCK (**d**) were measured by Western blot and quantified using GAPDH as the loading controls. Data are expressed as means ± SE, *n* = 5 for each group. * *p* < 0.05, ** *p* < 0.01 vs. NC; # *p* < 0.05, 5, ## *p* < 0.01 vs. MO; △*p* < 0.05 vs. PA

The qRT-PCR results showed that a significant increase of RhoA mRNA in the brain 20 and 40 dpi in the MO mice when compared with the NC mice (*P* < 0.01 or *P* < 0.05). The obvious decreased RhoA mRNA in the brain and spinal cord 20 and 40 dpi was shown in both PA and BSYSC mice as compared with the MO mice (*P* < 0.05, Fig. 1a). ROCK mRNA also appeared significant increase in brain and spinal cord 20 and 40 dpi in the MO mice as compared with the NC mice (*P <* 0.05). Decreased ROCK mRNA levels were observed in brain and spinal cord 20 and 40 dpi in BSYSC mice (*P* < 0.05 or *P* < 0.01), and it also decreased in brain 20 dpi and spinal cord 40 dpi in PA mice (*P <* 0.01, Fig. 7b).

Western blot analyses showed that RhoA protein level significantly increased, specifically in brain 40 dpi and in spinal cord 20 dpi in the MO mice as compared with the NC mice (*p* < 0.05 or *p* < 0.01). Decreased RhoA were observed in brain and spinal cord 20 and 40 dpi in BSYSC mice as compared with the MO mice (*P <* 0.05 or *P* < 0.01), RhoA was also decreased in brain 40 dpi and spinal cord 20 dpi in PA mice (*P <* 0.01). It was worth mentioning that the effect of BSYSC was better than that of PA (*P <* 0.05, Fig. 7c). ROCK protein was increased markedly in brain 20 dpi and spinal cord 40 dpi in MO mice as compared with the NC mice (*P <* 0.05). A significant reduction of ROCK protein was observed in brain 40 dpi in both PA and BSYSC mice as compared with the MO mice (*P <* 0.01), and ROCK was also decreased in spinal cord 20 and 40 dpi in BSYSC mice (*P <* 0.05, Fig. 7d).

## Discussion

In our previous studies, the characteristics of EAE mice were confirmed by the increase in neurological function scores, the existence of inflammatory infiltrates in brain and spinal cord, and damage to axons and the myelin sheath. It also appeared the immune imbalance in Th1/Th2 and Th17/Treg cells. However, BSYSC treatment could improve the clinical symptoms and neurological dysfunction, reduce the inflammatory response and regulate the cellular immunity. These findings indicated that BSYSC had the neuroprotective effects in EAE mice [22, 23, 25]. In the present study, we investigated the effects of BSYSC on reducing axonal injury and promoting axonal repair in EAE mice. We also explored further the regulation of BSYSC on NogoA/NgR and its signaling pathways RhoA/ROCK.

PGP9.5 is a specific ubiquitin-hydroxy-enzyme in nerve fibers, which widely distributed in neuron, nerve fibers and many kinds of neuroendocrine cells [26, 27]. It is transported through slow axoplasmic transport in axons of nerve cell, and is the highest content of soluble protein in neurons [28, 29]. Tau is a kind of neuron microtubule associated protein, which mainly concentrates in the axons [30–32]. It has the function of stabilizing the axonal microtubules and maintaining the axonal transport. The study found that PGP9.5 expression was reduced in rats after spinal cord injury [33]. The excessive Tau protein damaged the axoplasmic transport and caused the degeneration of neurons [34–36]. The abnormal phosphorylation of Tau protein was related with the absence of neurons and axons in chronic remission of recurrent EAE and secondary development of MS, leading to tissue degeneration, making disease progression to chronic stage [37]. In the current study, PGP9.5 protein expression was significantly decreased, but p-Tau was markedly increased in brain or spinal cord of EAE mice during acute and/or remission phase, indicating the axon and myelin sheath were seriously injured in the whole pathological process. After treatment of BSYSC, the levels of PGP9.5 protein were increased obviously, and p-Tau were decreased markedly, which indicated that the formula had the effect of reducing the nerve injury. This effect may be related to the reduction of immune inflammation. Indeed, our previous studies found that BSYSC regulated the ratio of CD4 + IL-17+/CD4 + CD25 + FoxP3+ T cells in the spleen, decreased the cytokines such as IL-17A, IL-6, IL-23 in the brain and mediated the balance of Th17/Treg cells [22, 23, 25].

GAP-43 is a kind of fast transport membrane protein, which is closely related to the neural development, axonal regeneration and synaptic reconstruction [38, 39]. It is found that GAP-43 is highly expressed in the axonal regeneration and synaptic growth after brain injury [40–42]. Therefore, GAP43 is used as a marker of axonal regrowth and synaptogenesis. Our results showed BSYSC increased the expression of GAP-43 in the brain and spinal cord of EAE mice, and the increase in GAP-43 with BSYSC thereby indicates its effects of promoting axon growth.

As a nuclear proliferation antigen, KI67 serves as a cell proliferation marker. Nestin is a marker of neural stem cells, which highly expresses in the mammalian neural precursor and is able to differentiate into neurons and glia cells. Various potentials of neural stem cells demonstrated including migrating to the damage regions to fill and replace impaired neural cells. Our results showed that KI67 and Nestin in the spinal cord were significantly enhanced after BSYSC treatment 40 dpi in EAE mice, revealing the underlying potential of BSYSC on enhancing neural stem cell proliferation. Neural stem cells can be differentiated into different phenotypes, repairing injured neurons, glial cells and axons [43, 44], thus promoting functional recovery in EAE mice. Whereas no statistical difference of KI67 and Nestin was found in the brain in this experiment, this might be related to the severity of the inflammation and demyelination in spinal cord of EAE mice [45].

A large number of studies have found that NogoA is a membrane protein in the CNS restricting neurite growth and synaptic plasticity via the extracellular domain (Nogo-66), it exerts the inhibitory activity by binding to a neuronal receptor complex containing NgR, p75NTR and LINGO-1 [46–48], and activates the downstream signaling pathways of RhoA/ROCK, acts on the actin cytoskeleton system to cause growth cone collapse and neurite outgrowth inhibition [49–51]. The studies have also demonstrated that NogoA/NgR and its RhoA/ROCK signaling pathways are involved in the development of MS or EAE. NogoA expression was markedly upregulated in surviving oligodendrocytes at the edge of chronic active demyelinating lesions of MS, whereas NgR was found to be significantly increased in reactive astrocytes and microglia/macrophages in these lesions [52]. TROY and LINGO-1 was also greatly enhanced in astrocytes and microglia in MS lesions [52, 53]. NogoA and RhoA are strongly expressed in brain tissues of MS and EAE rats [54, 55]. In this study, NogoA, NgR, RhoA and ROCK mRNA and protein expressions in the brain and/or spinal cord of mice were significantly increased at acute and/or remission stage. Our data were consistent with these above described findings [56]. It indicates that a large number of inhibitory factors were produced in the lesions of axon and myelin which activate RhoA/ROCK signaling pathways. Up to 40 days, the expressions of inhibitory factors were still increased, speculating that the degeneration of nerve existed in the whole progress of the disease. Therefore, blocking RhoA/ROCK signaling can reverse the inhibitory effects of these molecules on axon outgrowth and functional recovery. Some scholars use ROCK inhibitors such as Y-39983 and WAR-5 to treat EAE animal. Y-39983 attenuates EAE condition via inhibition of demyelination [57]. WAR-5 selectively suppresses the expression of ROCK II in spleen, brain and spinal cord of EAE mice, especially in spinal cord, accompanied by decreased expression of Nogo [58]. Here, we found that BSYSC can not only significantly reduce the nerve regeneration inhibitory factors NogoA and NgR but also inhibit its signaling pathways RhoA and ROCK. We also found that the changes of the above indexes were slight different in brain and spinal cords during the disease course, the explanation for this could be the formula treatment functioned more effectively at the early stage and tended to maximize the effects at 20 dpi or later, or simply due to the sensitivity of our detection methodology was not sufficient to resolve the potential differences quantitatively at this point.

Recent studies have found that many Chinese medical formulas have the effects of moderating NogoA/NgR [59]. Several herbs in BSYSC that we studied were also known to play important roles in inhibitory signal pathway. For examples, gastrodin, the main constituent of *Gastrodiae Rhizoma*, down-regulated the expression of the neuronal cytoskeleton remodeling-related negative regulators Slit1 and RhoA in the hippocampus of rat model of depression [60], San-Huang-Xie-Xin-Tang containing *Rhei Radix et Rhizoma* attenuated ROCK-II protein expression in U46619-induced primary pulmonary smooth muscle cells [61]; additionally, *Leonuri Herba*, *Scorpio* and *Gastrodiae Rhizoma* were shown have neuroprotective and neuroregenerative effect in vivo and in vitro [62–65]. Moreover, the molecules identified in BSYSC like forsythiaside, acteoside were reported for neuroprotective effects [66–71]. These findings strongly implied that the effects of BSYSC in MS or EAE animal model, could be cooperative action with multiple compounds, also involved cross components worked together for the common effects. The exact roles of the active components for treatment of EAE need to be further investigated.

## Conclusions

In summary, BSYSC exhibited the therapeutic effect on inducing neurogenesis and axon growth after axonal injury in EAE mice, which might be attributable to the modulation of NogoA/NgR and RhoA/ROCK. These findings provided new insights into the mechanism of BSYSC in the treatment of MS.

## Abbreviations

BSYSC: Bu Shen Yi Sui Capsule
CFA: Complete Freund’ adjuvant
CNS: Central nervous system
EAE: Experimental autoimmune encephalomyelitis
GTPase: Guanosine triphosphatase
IF: Immunofluorescence
MAG: Myelin-associated glycoprotein
MOG: Myelin oligodendrocyte glycoprotein
MS: Multiple sclerosis
NgR: Nogo-66 receptor
OMgp: Oligodendrocyte myelin glycoprotein
PA: Prednisone acetate
PTX: Pertussis toxin
QRT-PCR: Real-time quantitative reverse transcription-polymerase chain reaction; SE: standard error
TCM: Traditional Chinese medicine
